# Effects of miR-204-5p modulation on PAX6 regulation and corneal inflammation

**DOI:** 10.1038/s41598-024-76654-w

**Published:** 2024-11-02

**Authors:** Mojdeh Abbasi, Maryam Amini, Petros Moustardas, Quirin Gutsmiedl, Dina Javidjam, Shweta Suiwal, Berthold Seitz, Fabian N. Fries, Ava Dashti, Yedizza Rautavaara, Tanja Stachon, Nóra Szentmáry, Neil Lagali

**Affiliations:** 1 Division of Ophthalmology, Department of Biomedical and Clinical Sciences, Linköping University, 581 83 Linköping, Sweden; 2 Dr. Rolf M. Schwiete Center for Limbal Stem Cell and Congenital Aniridia Research, Saarland University, 66424 Homburg/Saar, Germany; 3 Department of Ophthalmology, Saarland University Medical Center, 66424 Homburg/Saar, Germany

**Keywords:** Aniridia, MiR-204-5p, PAX6, Keratitis, Corneal inflammation, Molecular biology, Eye diseases

## Abstract

Congenital aniridia is a rare eye disease characterized by loss of PAX6 protein leading to aniridia-associated keratopathy that significantly reduces vision. The miR-204-5p is a possible regulator of *PAX6* function and here we evaluate its effect in multiple in vitro and in vivo models. In vitro, miR-204-5p overexpression suppressed vascular factor *ANGPT1* in human limbal stem cells (T-LSC) and Pax6-knockdown LSC (mut-LSC), and in primary human limbal epithelial cells (LEC) at the gene and protein levels and following LPS stimulation. However, miR-204-5p inhibited *VEGFA* expression only in mut-LSCs and LPS-stimulated LEC. Also, miR-204-5p increased *PAX6* expression in mut-LSC and differentiated corneal epithelial cells, but not in LEC. Topical miR-204-5p after LPS-induced keratitis in mice failed to suppress *Vegfa*,* Angpt1*, *Il-1β*, and *Tnf-α* or rescue *Pax6* levels in contrast to in vitro results, although it significantly reduced the inflammatory infiltrate in the cornea. In *Pax6*^*Sey/+*^ aniridia mice, miR-204-5p did not rescue PAX6 levels or suppress *Vegfa*, *Angpt1*, or inhibit the ERK1/2 pathway. While short-term miR-204-5p treatment effectively suppresses *VEGFA* and *ANGPT1* and enhances *PAX6* expression in multiple corneal epithelia, effects are variable across primary and immortalized cells. Effects of longer-term in vivo treatment, however, require further study.

**Supplementary Information:**

The online version contains supplementary material available at 10.1038/s41598-024-76654-w.

## Introduction

Congenital aniridia is a rare pan-ocular disease that is in most cases a result of haploinsufficiency of the *PAX6* gene. *PAX6* is an essential transcription factor for regulating normal eye development and maintenance and is highly expressed in the iris, lens, and ocular surface epithelia such as the corneal and conjunctival epithelium^1^. A hallmark of congenital aniridia is the absence or severe underdevelopment of the iris, as well as foveal hypoplasia which often leads to visual impairment. Further vision impairment can occur due to secondary complications such as cataracts, glaucoma, and nystagmus^2^. Importantly, almost all aniridia patients also eventually develop an aniridia-associated keratopathy (AAK), characterized by progressive loss of corneal transparency, with conjunctival and vessel ingrowth that can lead to a total loss of vision^1^. AAK is also associated with compromised corneal wound healing and impaired corneal innervation, inflammation, and dry eye, which in turn lead to further vision loss and complications^3^. AAK is thought to develop as a consequence of limbal stem cell insufficiency and the breakdown of the limbal stem cell niche has been shown to lead to gradual AAK progression and eventually blindness^4^. Current AAK treatments aim to alleviate symptoms with varying approaches based on the degree of ocular surface injury. Conservative therapies such as ointments for maintaining tear film stability and autologous serum eye drops can aid in reducing corneal erosions and symptom burden, but often they provide only temporary relief and cannot slow the progression of the disease as they do not address the underlying PAX6 haploinsufficiency^5,6^. Similarly, surgical treatments such as limbal stem cell transplantation provide only temporary benefits and do not address the underlying inflammation and chronic wound healing in the cornea stemming from the haploinsufficiency^7^.

The *PAX6* gene produces the PAX6 protein whose dosage is tightly regulated at the pre-mRNA, posttranscriptional, or protein level. Of note, *PAX6* has been shown to undergo intricate posttranscriptional regulation by microRNAs^8^. MicroRNAs (miRNAs) are small non-coding RNA molecules that play pivotal roles as a posttranscriptional regulatory element of gene expression and may interfere with gene translation through adhering to a specific region in their target mRNAs^9^. For the *PAX6* gene, certain miRNAs have been identified that target its mRNA transcript. Given the high sensitivity of proper PAX6 dosage to its function, the binding of miRNAs to specific complementary sequences in the 3’ untranslated region (UTR) is pivotal for miRNA-mediated regulation of the *Pax6* mRNA^10^. This interaction typically results in translational repression or mRNA degradation, mediating post-transcriptional regulation of PAX6. Such precision regulation allows for maintaining optimal PAX6 expression levels, crucial for the delicate balance required during eye development and throughout adulthood^8^. Among the miRNAs, miR-204-5p has been proposed as one of the best-known miRNAs in negatively regulating gene expression levels through eye development in vertebrate species^11^. Notably, *PAX6* is identified to be one of the main targets of miR-204-5p^12,13^. Originating from the transient receptor potential cation channel subfamily M member 3 (TRPM3) gene in both humans and mice, the mature miRNA sequence which is derived from the 5’ arm of the precursor stem-loop sequence is known as miR-204-5p^14,15^. This strand is the major strand of mature miR-204, while miR-204-3p is the minor strand and presents at a much lower abundance^16^. In the human eye, miR-204-5p has been detected within various ocular tissues including the lens epithelium, trabecular meshwork, ciliary body, and corneal and conjunctival epithelial cells^6,17^. Dysregulation of miR-204-5p can have profound implications for ocular health; its mutations, together with mutations in *TRPM3* and *PAX6*, are implicated in various inherited eye disorders such as retinal dystrophy, iris coloboma, ocular neovascularization, and impaired corneal wound healing^18–20^. This further emphasizes the critical role of post-transcriptional miRNA-mediated regulation in modulating gene expression. Therefore, understanding the regulatory mechanisms of miRNA-204 holds promise for developing therapeutic interventions targeting ocular diseases with dysregulated gene expression^18,19,21^. Prior studies have explored the role of miR-204 in regulating *Pax6* genes under specific conditions, particularly highlighting the critical involvement of miR-204-5p in lens development which has been reported to be regulated by PAX6 ^13,15^.

In conjunctival impression cytology samples taken from patients with congenital aniridia, miR-204-5p was 65-fold downregulated relative to healthy controls, consistent with an invasive, inflammatory, and neovascular phenotype in AAK^17^. The conjunctival expression changes likely also affect gene expression of the neighboring limbal epithelial cells and differentiated corneal epithelial cells, which are highly perturbed in advanced AAK.

Although *PAX6* regulation by miR-204 has been reported in prior studies^12,13^, to date the effects of miR-204-5p on *PAX6* in ocular surface epithelia and how its downregulation affects the progression of limbal stem cell deficiency and inflammation leading to AAK, is still unknown. Also, given the lack of therapies targeting the underlying haploinsufficiency in congenital aniridia, it is of particular interest to evaluate whether compensating for the deficiency of miR-204-5p could lead to PAX6 normalization with beneficial downstream effects in the cornea. Therefore, we aimed to investigate the impact of miR-204-5p modulation on *PAX6* and downstream genes in ocular surface epithelia of various types, including primary limbal epithelial cells from healthy corneal donors, a cellular model of *PAX6* haploinsufficient human limbal epithelial stem cells and fully differentiated corneal epithelial cells. We also explored the role of miR-204-5p in cell and murine models of LPS-mediated corneal inflammation, and in a *Pax6*^*Sey/+*^ mouse model of aniridia.

## **Results**

### Endogenous miR-204-5p expression is elevated in the murine cornea relative to human corneal epithelial cell lines

**Fig. 1:**
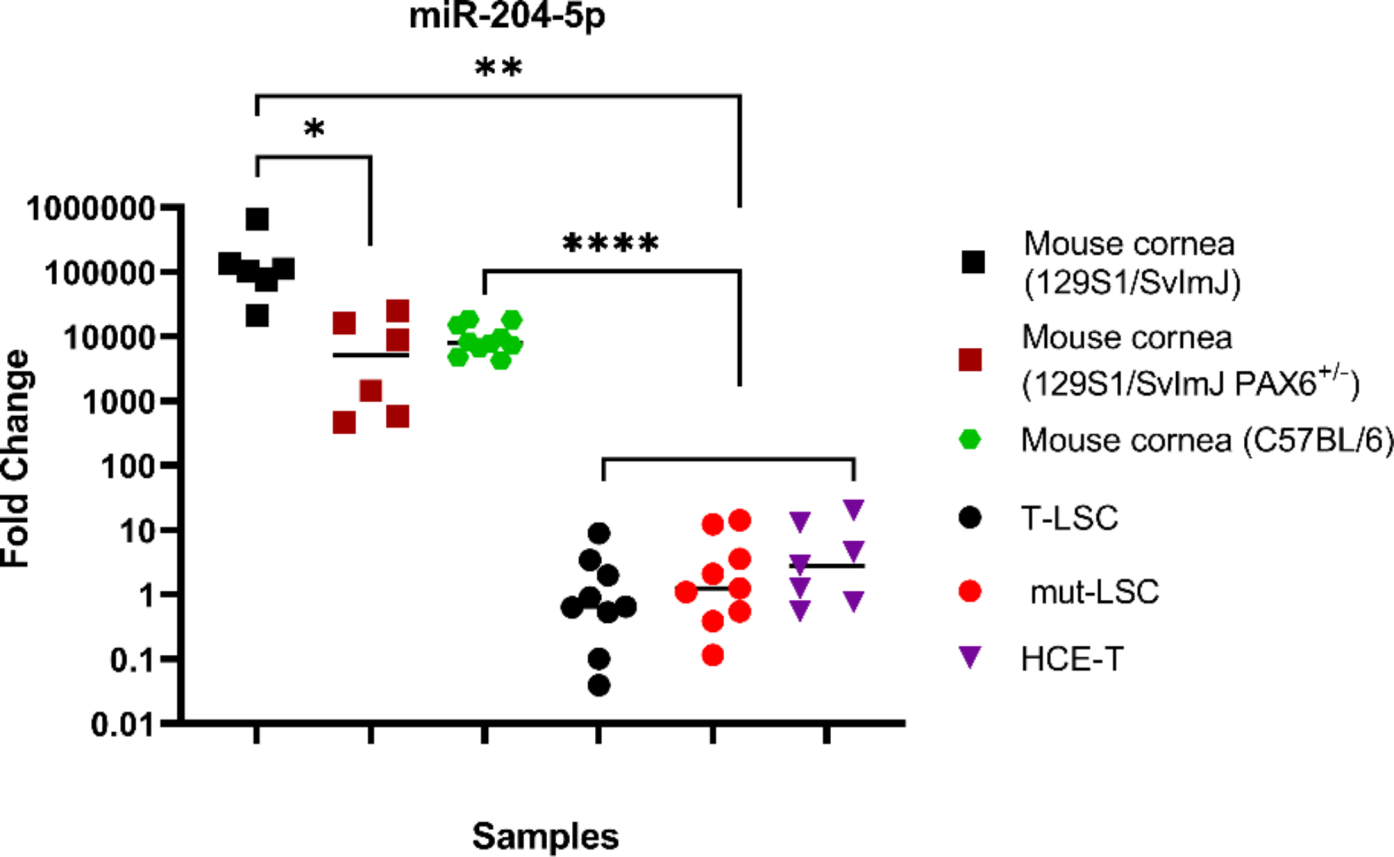
Comparative analysis of miR-204-5p expression levels in human corneal epithelial cell lines and mouse corneas on different backgrounds. qRT-PCR analysis revealed significantly lower miR-204-5p expression in T-LSC, mut-LSC, and HCE-T relative to the corneas of C57BL/6 (*p* < 0.0001) and 129S1/SvlmJ strains (*p* = 0.005 for T-LSC and mut-LSC and *p* = 0.009 for HCE-T). Corneas of the WT-129S mice showed higher levels of miR-204-5p relative to the *Pax6*^+/−^ corneas of littermates (*p* = 0.02).

Given prior results in primary human conjunctival cells that miR-204-5p was highly suppressed in aniridia^17^, we aimed to assess the endogenous expression of miR-204-5p within various human corneal surface epithelial cell lines as well as directly within the mouse cornea. miR-204-5p expression was found to be relatively low in all corneal epithelial cell lines, including differentiated HCE-T, T-LSC, and mut-LSC cell lines, without any change noted across the cell lines (Fig. 1, *p* = 0.31). WT mouse corneas with C57BL/6 background, however, had significantly higher levels of this miRNA relative to all cell lines (*p* < 0.0001). When compared to mouse corneas of 129S1/SvlmJ background (either WT or Het-*Pax6*^*Sey*/+^), similarly enhanced levels of miR-204-5p were noted relative to all three cell lines (*p* = 0.005 for T-LSC and mut-LSC, *p* = 0.009 for HCE-Ts). Comparing the level of miR-204-5p in corneal tissue from mice of 129S1/SvlmJ background revealed that WT-129S corneas exhibited a 23-fold enhancement relative to Het mice on the same background (*p* = 0.02).

### miR-204-5p enhances *PAX6* and suppresses vascular factors in LSC cell lines

**Fig. 2:**
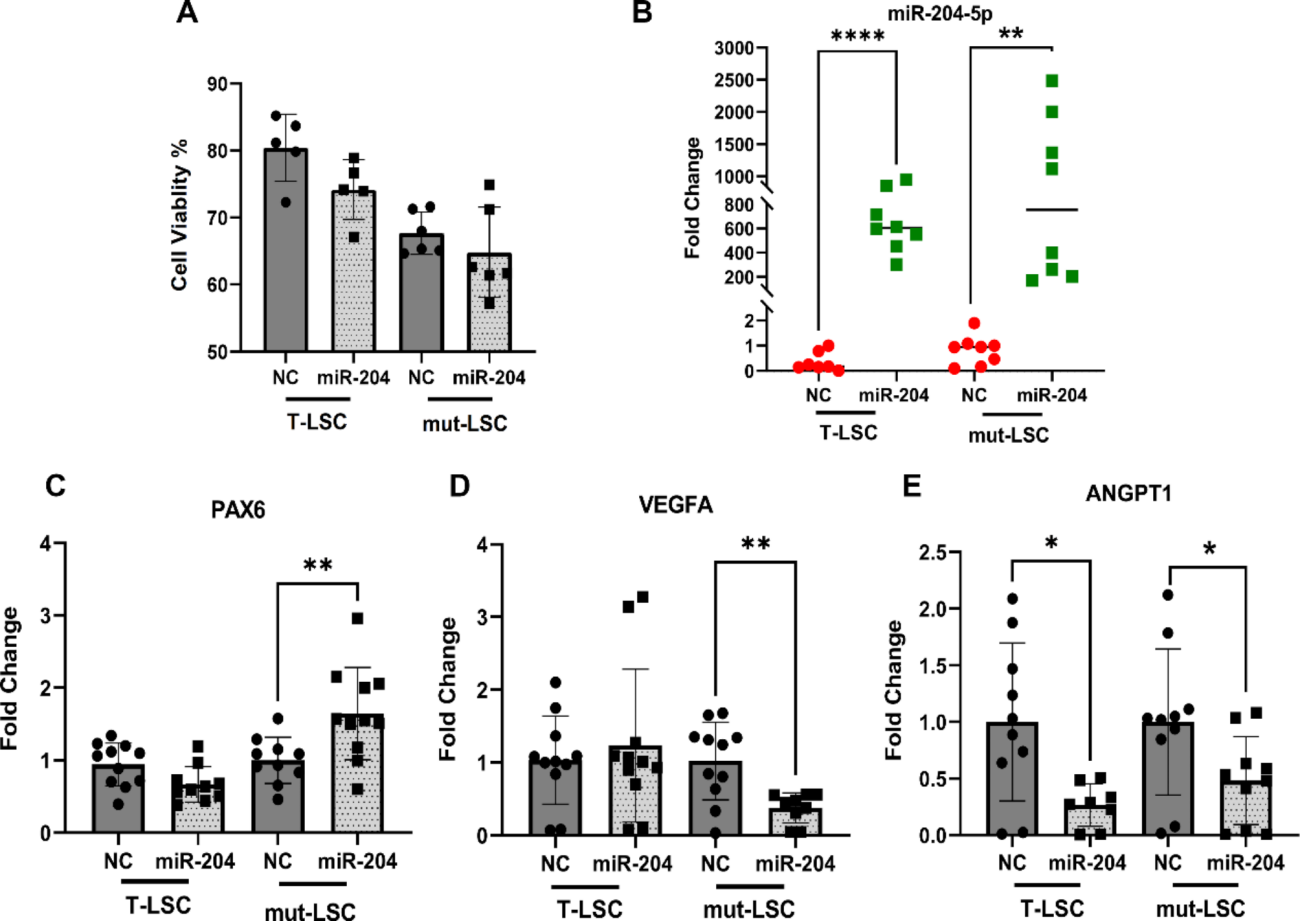
miR-204-5p administration in T-LSC and mut-LSC modulates levels of *PAX6* and canonical vascular growth factors. (**A**) Live/dead cytotoxic assay showed no cytotoxicity of miR-204-5p in T-LSC and mut-LSC cells. Data are presented as mean ± SD of the percentage of survival. (**B**) miR-204-5p was significantly increased in both T-LSC (*p* < 0.001) and mut-LSC (*p* < 0.01) 24 h after transfection. (**C**) *PAX6* expression was significantly elevated in mut-LSC following miR-204-5p transfection (*p* = 0.007) but not in T-LSC (*p* = 0.02). (**D**,**E**) Increased levels of miR-204-5p stimulated downregulation of *VEGFA* (*p* = 0.003) and *ANGPT1* (*p* = 0.04) in mut-LSC, while *ANGPT1* expression also declined in T-LSC (*p* = 0.01). miR-204 denotes miR-204-5p in the graph labels.

First, the potential cytotoxicity of exogenous miR-204-5p on LSC was examined. T-LSC and mut-LSC were treated with either the miR-204-5p mimic or the scrambled negative control mimic (NC) for 24 h. The live/dead assay did not reveal any cytotoxicity and no significant differences were observed within cells treated with either the mimic or NC (Fig. 2A).

To investigate if miR-204-5p modulation could influence *PAX6* expression, we first examined the level of miR-204-5p post-transfection, verifying its upregulation in T-LSC and mut-LSC treated with miR-204-5p compared to NC (Fig. 2B). Evaluation of *PAX6*, *VEGFA*, and A*NGPT1* expression 24 h following transfection showed 1.6-fold increase in PAX6 expression in mut-LSC relative to NC (*p* = 0.007) but not in T-LSC (Fig. 2C). miR-204-5p treatment also led to significant reductions in the canonical vascular growth factors and stabilizers *VEGFA* and *ANGPT1* that was apparent in mut-LSC, while only *ANGPT1* expression was suppressed in T-LSC (Fig. 2E).

### miR-204-5p upregulates *PAX6* in HCE-T

**Fig. 3:**
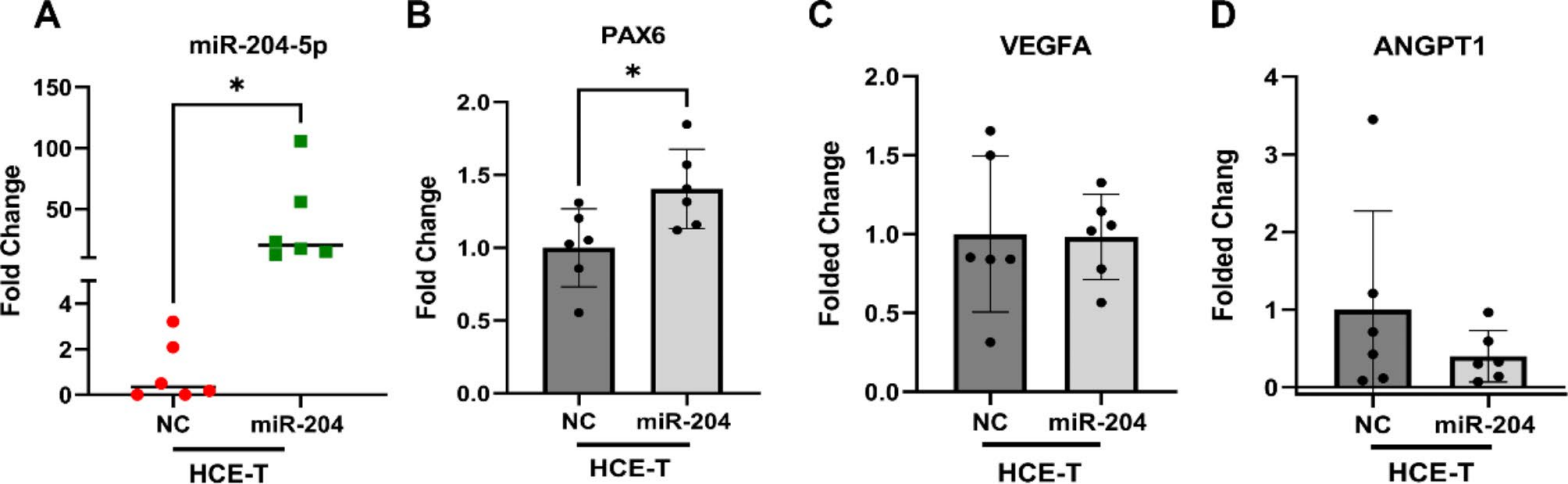
miR-204-5p modifications resulted in *PAX*6 higher levels in HCE-T cells. (**A**) There was a statistically significant level of miR-204-5p 24 h post-transfection in this non-limbal corneal epithelial cell line (*p* = 0.03). (**B**) qRT-PCR verification indicated an increased level of *PAX*6 following the cells being treated with miR-204-5p (*p* = 0.02). miR-204 denotes miR-204-5p in the graph labels.

To evaluate whether miR-204-5p could induce similar effects in *PAX6* and vascular mediators in non-limbal (differentiated) corneal epithelial cells, HCE-T cells were transfected with miR-204-5p for 24 h. miR-204-5p levels were significantly elevated, but by an order of magnitude lower than in T-LSC and mut-LSC (Fig. 3A). However, transfection resulted in significantly elevated *PAX6* expression by a factor of 1.4 mirroring the effect on mut-LSC (Fig. 3B). The suppressive effect on *VEGFA* and *ANGPT1*, however, was not detected (Fig. 3C,D).

### miR-204-5p partially regulates gene and protein expression in primary LEC

**Fig. 4:**
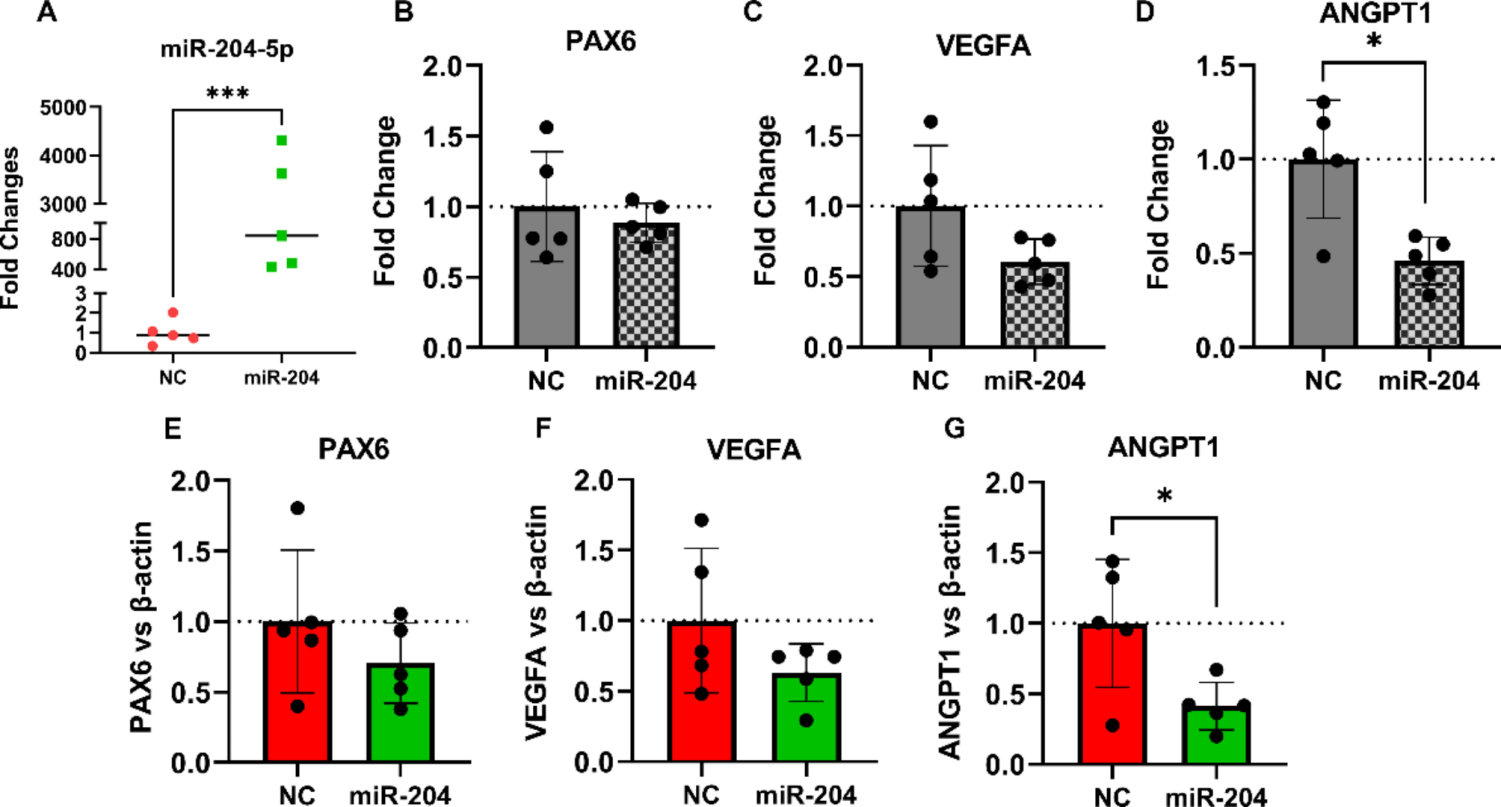
miR-204-5p modulation of gene and expression in primary LECs. (**A**) miR-204-5p level significantly elevated following the transfection (*p* < 0.001). (**B**–**D**) While mRNA expression levels of *PAX6* and *VEGFA* did not show remarkable alterations (*p* = 0.08, 0.16, respectively), *ANGPT1* was significantly downregulated (*p* = 0.03) following transfection with miR-204-5p in primary LEC. (**E**–**G**) A similar trend was observed in protein levels via western blot of PAX6 (*p* = 0.14), VEGFA (*p* = 0.13), and ANGPT1 (*p* = 0.03) in primary LEC following miR-204-5p transfection. miR-204 denotes miR-204-5p in the graph labels.

To determine if miR-204-5p could also induce gene regulatory effects in primary epithelial cells, primary LEC were transfected with miR-204-5p mimic or NC. miR-204-5p was significantly upregulated following transfection (*p* < 0.001, Fig. 4A). Unlike in the epithelial cell lines, *PAX6* mRNA expression was not affected by miR-204-5p overexpression in primary LECs (*p* = 0.08) (Fig. 4B) nor was VEGFA (*p* = 0.16, Fig. 4C). Similar to the cell lines, however, miR-204-5p significantly suppressed ANGPT1 expression (*p* = 0.03, Fig. 4D).

To determine changes induced by upregulation of miR-204-5p at the protein level, western blot analysis was performed on total proteins from the primary human LEC. Protein-level changes followed the pattern of mRNA change, with PAX6 and VEGFA proteins not significantly altered (*p* = 0.14 and *p* = 0.13 respectively. Figure. 4E,F) while a significant reduction was found for ANGPT1 (*p* = 0.03, Fig. 4G).

### Regulatory effects of miR-204-5p in LPS-stimulated primary LEC

**Fig. 5:**
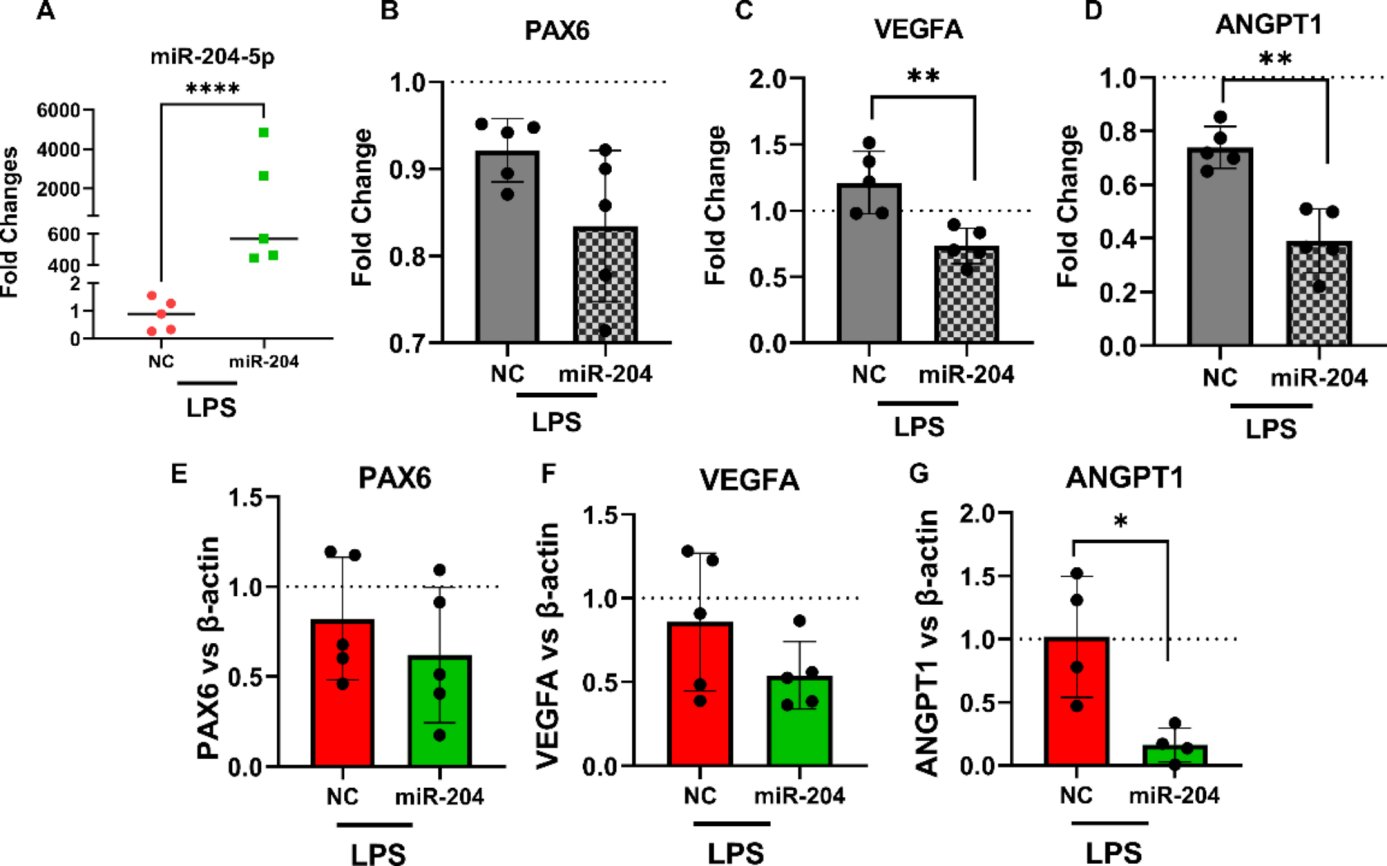
Effect of miR-204-5p treatment in LPS-stimulated primary LEC. (**A**) miR-204-5p showed significantly higher levels following transfection (*p* < 0.0001). (**B**–**D**) mRNA expression levels of *VEGFA* and *ANGPT1* indicated a significant decline after being treated with miRNA in the LPS condition (*p* = 0.006 and *p* = 0.004, respectively) while the *PAX6* level remained constant. (**E**–**G**) Protein expression analysis revealed a significant decrease in ANGPT1 (*p* = 0.01) after miRNA treatment in the LPS condition while VEGFA (*p* = 0.052) and PAX6 (*p* = 0.48) did not present significant alterations. miR-204 denotes miR-204-5p in the graph labels.

To determine whether the observed regulatory effects of miR-204-5p would persist in the context of the inflammation typically present in the cornea in aniridia, LEC transfected with NC or miR-204-5p were challenged with LPS. Evaluating the level of miR-204-50 indicated significantly higher levels of this miRNA after transfection (*p* < 0.0001, Fig. 5A). As observed in non-stimulated LEC, *PAX6* expression was not significantly altered at the mRNA or protein level (Fig. 5B,E) (SFig. 1). Both *VEGFA* and *ANGPT1*, however, were suppressed at the mRNA level (*p* = 0.006, *p* = 0.004, respectively) (Fig. 5C, D) and at the protein level, a similar pattern was observed, with *ANGPT1* (Fig. 5G) (SFig. 1) suppression (*p* = 0.01) and tendency towards VEGFA suppression (*p* = 0.052, Fig. 5F) (SFig. 1).

### miR-204-5p imparts partial suppression of inflammatory corneal infiltrate in an acute model of LPS-induced keratitis

**Fig. 6:**
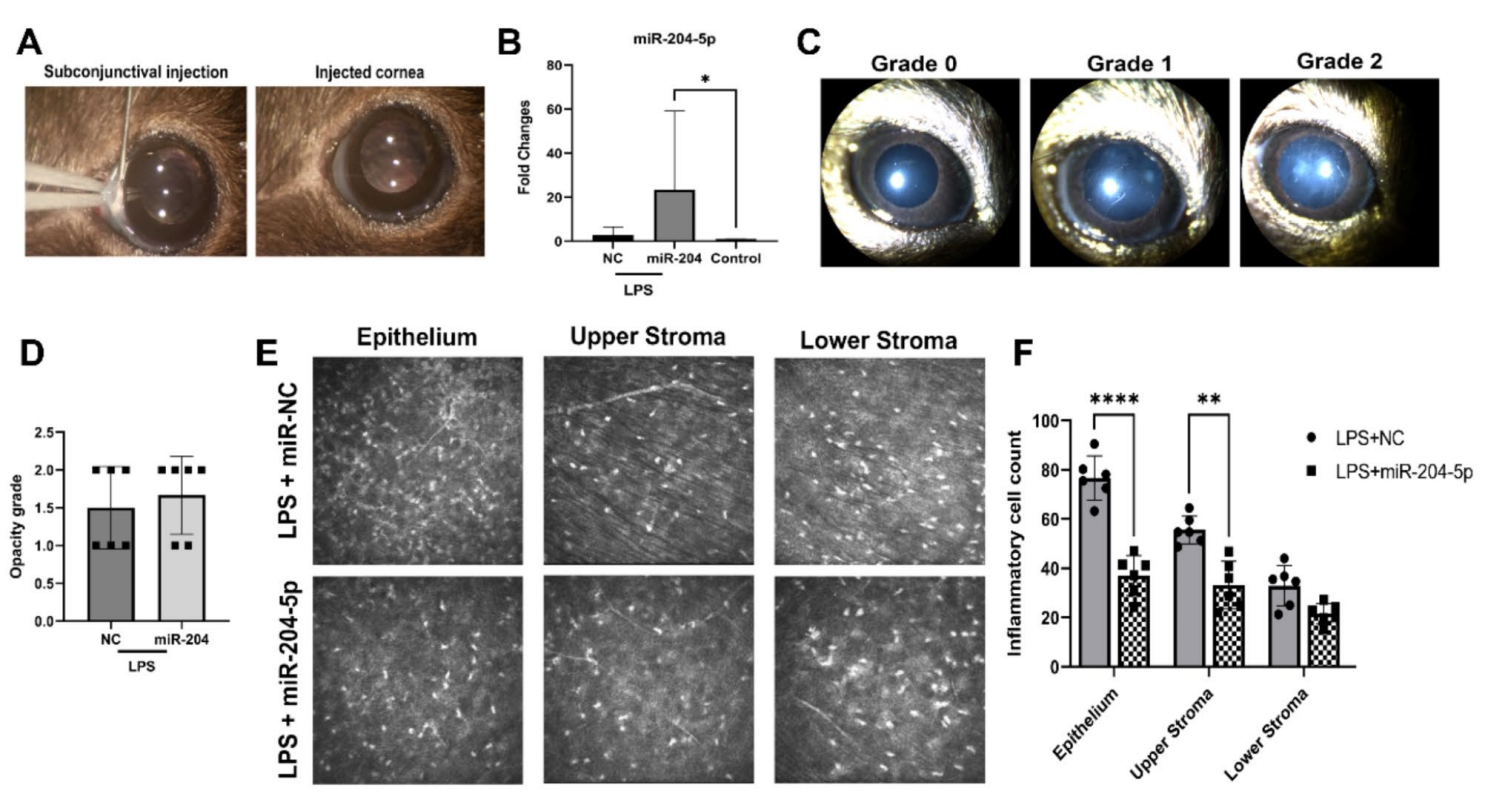
miR-204-5p treatment contributed to partial in vivo amelioration of LPS-mediated corneal keratitis. (**A**) subconjunctival co-injection of miRNA and LPS. (**B**) 48 h after LPS induction and after QID miR-204-5p topical treatment, corneal tissues had a significantly elevated level of miR-204-5p relative to the non-treated control group (*p* = 0.03). (**C**) Representative slit-lamp images from different clinical opacification grades were observed among both treated groups. (**D**) No significant alteration in corneal transparency was noted among experimental groups 48 h post-induction (E, F) LPS injection stimulated an inflammatory infiltrate within corneal layers and miR-204-5p treatment significantly reduced the cell infiltration in the epithelium and anterior stroma (*p* < 0.0001, *p* < 0.01 respectively).

LPS-induced keratitis is characterized by an intense level of corneal inflammation consisting mainly of neutrophil infiltration which ultimately results in damage to the corneal tissue, loss of transparency, and in severe stages, vessel ingrowth^22^. Using this as a model for corneal inflammation, mouse corneas were initially co-injected with LPS and miR-204-5p subconjunctivally (Fig. 6A) and treated topically for two days with miR-204-5p. Within the corneal tissue, treatment enhanced the miR-204-5p level compared to non-treated control corneas (*p* = 0.03, Fig. 6B). Following miR-204-5p administration, no abnormal alterations were discerned upon slit lamp examination, with LPS induction resulting in a clinical opacification score of 1–2 two days after injection; this score did not significantly differ between the treatment groups (Fig. 6C,D). Despite the absence of discernable differences in corneal transparency, the substantial invasion of hyper-reflective inflammatory cells (identified in previous studies as neutrophil granulocytes^23,24^) in the central cornea 2-days after LPS induction was diminished with miR-204-5p treatment by almost 2-fold in the epithelium (*p* < 0.0001) and 1.5-fold (*p* < 0.01) in the anterior stroma as evidenced by IVCM (Fig. 6E,F).

**Fig. 7:**
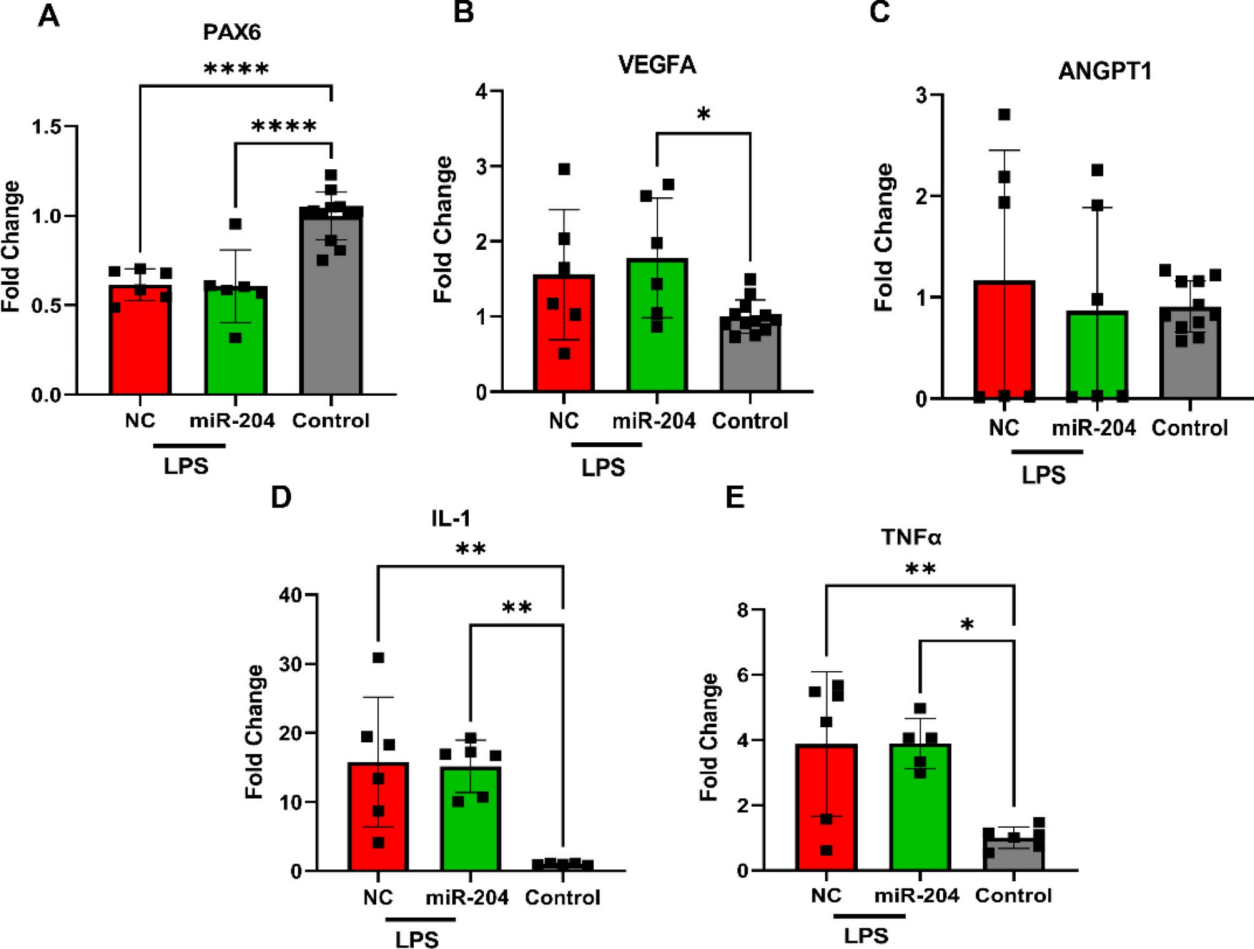
Effect of short duration miR-204-5p treatment in an acute model of LPS-induced keratitis. (**A**) A decline in *Pax6* mRNA level (*p* < 0.0001) following LPS-induced keratitis in mice cornea was observed, but miR-204-5p treatment for 48 h failed to mitigate this decline. (**B**,**C**) Following LPS stimulation, *Vegfa* mRNA increased (*p* = 0.04) while a non-significant increasing trend in *Angpt1* levels was also noted, with neither being mitigated by miR-204-5p treatment. (**D**,**E**) Despite observing significant upregulation in inflammatory factors *Il-1β* (*p* = 0.003) and *Tnf-α* (*p* = 0.008) following LPS-induced keratitis, miR-204-5p treatment for 48 h did not suppress these levels. (miR-204 denotes miR-204-5p in the graph label).

Subsequently, we assessed the expression levels of *Pax6*,* VegfA*, and *Angpt1* in the same corneas. qRT-PCR analysis revealed that LPS induction noticeably diminished *Pax6* expression (Fig. 7A) while concurrently inducing a substantial increase in *VegfA* but not in *Angpt1* (Fig. 7B,C). Nevertheless, despite miR-204-5p treatment, this alteration remained largely unaffected 48 h after treatment. Additionally, while LPS induction significantly increased the expression of the pro-inflammatory markers *Il-1β* and *Tnf-α* relative to untreated controls (*p* = 0.003 and *p* = 0.008, respectively), miR-204-5p treatment for 48 h did not significantly affect these levels (Fig. 7D,E).

### A short course of topical miR-204-5p treatment does not enhance *Pax6* expression or suppress vascular mediators in a mouse model of aniridia

**Fig. 8:**
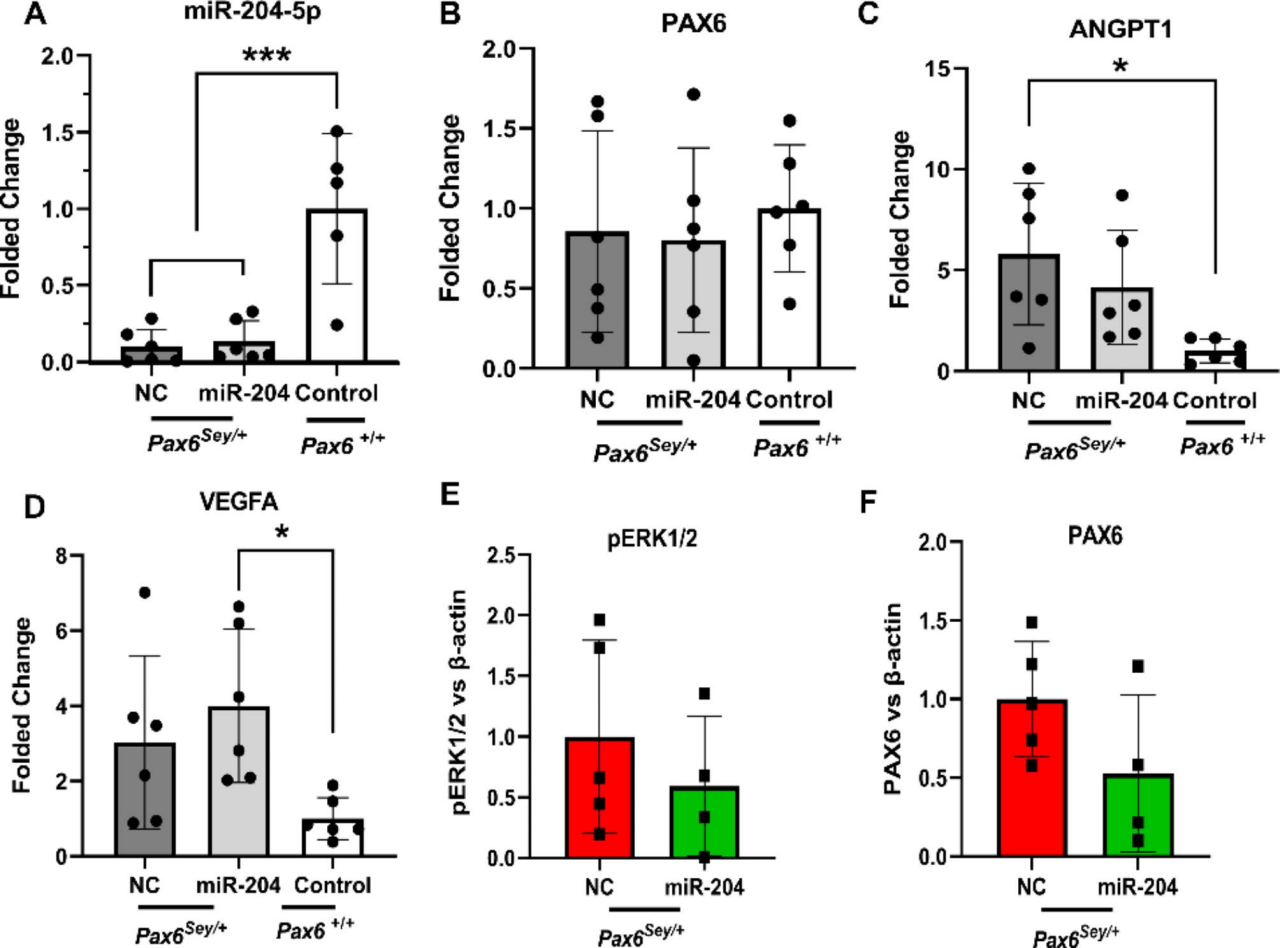
Effects of short-duration miR-204-5p treatment at the mRNA and protein level in the *Pax6*^*Sey*/+^ aniridia mouse model. (**A**) miR-204-5p levels were considerably downregulated in the corneas of the *Pax6*^*Sey*/+^ aniridia mice relative to WT-129S mice with similar backgrounds (*p* < 0.001) but this was not altered by short-duration miR-204-5p treatment. (**B**) miR-204-5p treatment correspondingly did not change *Pax*6 mRNA expression (*p* = 0.8). (**C**,**D**) In *Pax6*^*Sey*/+^ mice, *Vegfa* and *Angpt1* markers were significantly upregulated compared to WT-129S controls while miRNA treatment similarly did not change these levels (*p* = 0.02 and *p* = 0.01, respectively). (**E**) Following miR-204-5p treatment in the model, pERK1/2 protein was not suppressed (*p* = 0.4). (**F**) Accordingly, the PAX6 protein level was unchanged (*p* = 0.1). miR-204 denotes miR-204-5p in the graph labels.

One of the important hallmarks of aniridia in patients is the invasion of inflammatory cells into the cornea^7,25^. Given that miR-204-5p was highlighted in previous studies to post-transcriptionally regulate *ANGPT1 *and has been proposed as an interesting candidate for the treatment of inflammatory corneal neovascularization^26,27^, we also aimed to evaluate the effect of miR-204-5p treatment in a *Pax6*^*Sey/+*^mouse model^28^. Examining the level of miR-204-5p expression revealed a lower level of miR-204-5p in *Pax6*^*Sey/+*^ mice treated with either miR-204-5p or NC compared with their WT-129S littermates (consistent with results in Fig. 1). These data however, indicate a lack of miR-204-5p enhancement in response to miR-204-5p treatment in the corneal tissue (Fig. 8A).

Accordingly, mRNA expression levels of *Pax6* did not change following short-term miR-204-5p treatment (Fig. 8B). Also, as expected, vascular mediators *VegfA* and *Angpt1* were generally increased in *Pax6*^*Sey/+*^ mice relative to WT-129S littermates (Fig. 8C,D), but again in accordance with lack of miR-204-5p enhancement in the tissue, these levels did not change following short-term miR-204-5p treatment (Fig. 8C,D).

We also investigated whether miR-204-5p can target the ERK pathway in our mouse model, given its prior association with the indirect restoration of PAX6 expression in mut-LSC cells^29,30^.

Western blot analysis of phosphorylated ERK1/2 (pERK1/2) protein in the same corneal tissues following short-term miR-204-5p administration revealed a corresponding lack of significant suppression of pERK1/2 and lack of enhancement of downstream PAX6 protein levels (Fig. 8E,F, SFig. S2A). This finding was also consistent with the lack of rescue of *Pax6* at the mRNA level (Fig. 8B). Examining the ocular surface in vivo by slit lamp microscopy did not reveal noticeable alteration in the ocular surface during the short-term 7-day duration of the experiment. A variable grade of corneal opacity was present and remained unchanged within both experimental groups (SFig. S2B) and no significant alteration was observed in the corneal opacity prior to and after the miR-204-5p or NC treatment (SFig. S2C).

## Discussion

In the present study, we aimed to explore the therapeutic potential of miR-204-5p using multiple corneal epithelial cell types and murine models, with a focus on attenuating the proinflammatory and proangiogenic features of AAK typically detected in aniridia patients^17^ by addressing the underlying *PAX6* haploinsufficiency. Our findings offer insights into the potential of miR-204 in alleviating ocular surface complications both in aniridia and in other pathologic contexts.

In this study for the first time, we demonstrated that *PAX6* levels in mut-LSC and HCEC could be significantly upregulated by miR-204-5p treatment leading to a significant reduction in proangiogenic *VEGFA* and *ANGPT1*, while miRNA administration indicated no deleterious effects on cell viability. Moreover, miR-204-5p topical application on the cornea in the LPS-induced acute keratitis model partially mitigated inflammatory cell infiltration in the cornea after only 48 h, further highlighting the therapeutic potential of miR-204 in the context of ocular inflammatory disease.

Recent research has shed light on the role of miRNAs in the maintenance of corneal homeostasis^9^, among which miR-204 has attracted interest owing to its abundant expression within various ocular tissues^6,15,17,31^. Here we found elevated expression of miR-204-5p in murine corneal tissues in comparison to epithelial stem cells and HCEC, corroborating earlier findings of tissue-specific expression of this miRNA and its presumed role in regulating ocular development and maintaining proper ocular function^18,19,21^. We also detected markedly diminished miR-204-5p levels in the corneal tissues of *Pax6*^*sey*/+^mice compared to the WT-129S. This corresponds with a recent study on human aniridia patients indicating dysregulation of miR-204-5p expression in conjunctival cells in aniridia, exhibiting a roughly 65-fold decline in expression levels^17^. An earlier study also noted a dramatic change in miR-204-5p level of up to 200-fold in a murine model of corneal wound healing, further supporting the notion that miR-204 is abnormally suppressed in corneal pathologies^6^.

Previous studies have shown that loss of *PAX6* function results in altered levels of numerous genes, the majority of which are secondary targets to *PAX6 *implying that this gene either directly or indirectly regulates the expression of genes in specific miR-204-5p pathways^12^. Based on that, our study aimed to assess the impact of miR-204-5p on its previously known targets including ANGPT1 and VEGFA, in both normal and *PAX6*-mutated LSC. *VEGFA* and *ANGPT1 *play pivotal roles in angiogenesis whether physiological or pathological, and upon stimulation under inflammatory conditions, these factors orchestrate the expression of inflammatory cytokines^32^. ANGPT1 along with VEGFA have been shown to be expressed during inflammatory processes such as wound healing^26,33 ^and both markers have been reported to be directly targeted by miR-204 through 3’-UTR-dependent inhibition^19,26,32^.

In this study, *VEGFA* and *ANGPT1* expression were suppressed by 70% and 50% respectively, after miR-204-5p treatment in mut-LSC (Fig. 2D,E), which was associated with significantly elevated levels of *PAX6* in these cells. This finding not only proposes a novel co-regulatory system where *PAX6* can control the regulation of these genes in a miR-204-mediated manner but also highlights the potential of miR-204-5p in upregulating *PAX6* levels in cells that originally exhibit lower levels of *PAX6* expression due to CRISPR-Cas9-induced mutation.

VEGFA has also been previously noted to be elevated in aniridia patients^17^. Nevertheless, in T-LSC with a normal *PAX6* expression pattern, only *ANGPT1* was reduced whereas *PAX6* and *VEGFA *remained constant after miR-204-5p treatment. This is in agreement, however, with a model of spontaneous corneal neovascular dystrophy, where ANGPT1 but not VEGFA was shown to mediate corneal neovascularization that correlated with loss of miR-204 expression^19^, suggesting that anti-VEGF therapy alone is insufficient. These discrepancies may arise because miR-204-5p performs its actions through various molecular pathways and therefore combinatory therapies might be a more efficient technique against pathological conditions mediated by it. In HCEC, we also found an approximately 40% higher level of PAX6 subsequent to miR-204-5p treatment, providing further evidence that *PAX6* is part of the miR-204-5p transcriptional regulatory network. These findings are also in agreement with a study reporting that *Trpm3* and its intronic miR-204-5p are co-regulated by *PAX6* during ocular development and that in the postnatal mouse eye, the expression pattern of this miRNA resembles that of PAX6^13^. This mechanism is similarly supported by findings of *PAX6* directly targeting *Trpm3*^12 ^although TRPM3 transcript was concomitantly found to be reduced in aniridia patients^17^.

Similarly, in primary cells, miR-204-5p treatment either with or without LPS treatment suppressed ANGPT1 at the mRNA and protein level while for VEGFA it was only observed to be significantly suppressed by miR-204-5p after the primary cells were stimulated by LPS. However we did not observe such effects of miR-204-5p on these markers in HCECs and therefore further studies are needed to determine the source of the differences noted. Nevertheless, our results are partially consistent with a previous study reporting that overexpression of miR-204-5p partly reversed the LPS-induced *ANGPT1* expression in endothelial cells, further verifying the post-translational regulation of this miRNA on *ANGPT1* while LPS enhanced the stability of *ANGPT1* by reducing the abundance of miR-204^26^.

In vivo, using an acute model of LPS-induced keratitis we demonstrated that despite unaltered inflammatory markers at the mRNA level, miR-204-5p suppressed inflammatory cell infiltration into the cornea in the acute phase, leaving the cornea with an average of 55% reduced levels of inflammatory cells compared to those treated with vehicle. Other studies supported this anti-inflammatory role with suggested functions of this miRNA in maintaining the epithelial barrier function and normal cell physiology^26,34^. In another study, Zhou et al. administered miR-204-5p-containing exosomes as eye drops to patients suffering from Graft-versus-host disease-associated dry eye disease which resulted in significant attenuation of inflammation and improved epithelial recovery with relief of symptoms^35^.

Inflammatory conditions are generally accompanied by intense angiogenesis and a multitude of inflammatory cytokines, including TNFα and IL-1 that are released by inflamed tissues^26^. Correspondingly we noticed aggravated levels of these markers following LPS inflammatory stimulation in the mice corneas which could not be alleviated at the molecular level by miR-204 treatment (Fig. 7D,E). This may be attributed, in part, to the tendency of each distinct miRNA (here miR-204-5p) to typically have a modest or restricted influence on the protein production of its targeted transcript^36^, and therefore the in vivo effects observed here might entail the effects of multiple miRNAs and their influences, suggesting that in many cases miRNAs together finely tune the functional output^8,37^. This complexity presents a challenge in developing therapeutic interventions focused on targeting an individual miRNA. The anti-inflammatory role of miR-204 has been recently elucidated in rats with diabetic retinopathy where the effect was likely mediated through Bcl-2 and SIRT1 upregulation, thus providing insight into another potential mechanistic pathway underlying this condition^38^. However, given the efficacy of miR-204-5p in reducing inflammation and promising in vitro results, it is recommended that future studies consider extended treatment durations and possibly higher concentrations which might elicit stronger and more beneficial effects.

Analysis of PAX6 expression in the corneal tissue of miRNA-treated *Pax6*^*Sey*/+ ^mice showed no restoration of PAX6 level which could be attributed at least in part, to the brief duration of treatment relative to the severity of aniridia-related complications evident in this model such as substantial opacity and vascularization^39^. Alternatively, the result may be due to inherently lower miRNA levels in the corneas of *Pax6*^*Sey*/+^ mice compared to wildtype (WT-129S) (Fig. 1), which could not be sufficiently enhanced by short-term topical treatment with one reason being the compromised ocular surface epithelial cells in the aniridia corneas^40^. The inflammatory markers such as *ANGPT1* and *VEGFA* exhibited a significant upregulation of around 5-fold in the corneas of *Pax6*^*Sey*/+^ mice compared to corneas of WT-129S counterparts which despite the miRNA administration, remained unabated thereafter. Similarly, the heightened opacity and pronounced vascularization observed in the aniridia mice corneas remained unaltered post-treatment, and no discernible changes were discovered despite the 7 days of topical miRNA treatment.

We also examined whether miR-204-5p can affect the ERK pathway in our mouse model. A previous report showed that activating MEK/ERK pathways could strongly reduce PAX6 production and showed that inhibiting this pathway in vitro could be considered as a therapeutic strategy to rescue the PAX6 levels^29,30^. Given the lack of miR-204-5p enhancement and thereby lack of effects downstream in the corneal tissue following treatment with the mimic in the *Pax6*^*Sey/+ *^model, we investigated whether the lack of effect was also consistent with the lack of inhibition of the MEK/ERK pathway.

However, our study showed that miR-204-5p did not significantly inhibit the ERK pathway, emphasizing the concurrent lack of increased PAX6 levels we observed. These results underscore significant dysregulation of these factors in aniridia conditions, necessitating further investigation of miR-204-5p in relevant mouse models to clarify its impact in such conditions.

An important limitation of our study is the short timeframes used for treatment, a fact that was only apparent after assessing the results of the experiments. Additionally, the acute-phase nature of the LPS model may not fully mirror the chronic inflammation seen in conditions like AAK. However, the brief duration of miRNA treatment was sufficient to detect effects in vitro, and could partially protect against heightened inflammation in vivo, suggesting the need for further studies with prolonged treatment durations to comprehensively explore these dynamics, particularly in slow-developing models of corneal pathology.

Overall, our study provides valuable insights into the regulatory effects of miR-204-5p on various corneal epithelial cells and offers opportunities for examining its therapeutic potential in the context of inflammatory conditions of the cornea. The dysregulation of miR-204-5p and its downstream targets emerge as a potential therapeutic axis for aniridia and PAX6-related conditions, while interventions aimed at its restoration hold promise in rebalancing PAX6 levels.

## Materials and methods

### Primary limbal epithelial cell isolation

With approvals from the Ethical Committee of Saarland (Protocol No. 21/21), primary limbal epithelial cells (LEC) were isolated from corneoscleral rims of the Klaus Faber Center for Corneal Diseases including Lions Eye Bank, following their use for corneal transplantation. In short, the limbal area was punched using a 1.5 mm punch (Kai Medical, Solingen, Germany) and incubated with 0.5 mg/ml Collagenase A (Roche Pharma AG, Basel, Switzerland) overnight. The day after, the cell suspension was filtered through a 20 μm Cell Trics filter. The cells that were attached to the filter were washed out, using 3 ml trypsin-EDTA-solution (Sigma-Aldrich GmbH, Deisenheim, Germany). After 10 min of incubation with trypsin at 37ºC and 5% CO2, Dulbecco’s Modified Eagle Medium (DMEM) containing 10% FCS was added^41–43^. Cells were then centrifuged at 200 g for 5 min and cultured in a Keratinocyte growth medium (KSFM) (Cat. Nr. C-20111, Promocell, Germany).

### Telomerase-immortalized limbal stem cell (T-LSC), CRISPR/Cas9 mutated PAX6^+/−^ LSC (mut-LSC), and human corneal epithelial cell-transformed (HCE-T) cell lines

T-LSC and mut-LSC cell lines were obtained as a gift from D. Aberdam, (INSERM, Paris) and have been described previously^44–46^. Cells were cultured in Keratinocyte Growth Medium 2 (C-20211, Merck) supplemented with 25 µg/mL bovine pituitary extract (BPE; 13028014, Life Technologies from Thermo-Fisher) 0.2 ng/mL recombinant human epidermal growth factor (EGF; Peprotech AF-100-15, from Gibco™), 2mM Glutamine (A2916801, Gibco™), 0.4mM CaCl_2_(21115, Merch), and 100 U/mL Penicillin/streptomycin (15140122, Gibco™ from Life Technologies- Thermo-Fisher). HCE-T cell line was purchased from RIKEN BRC Cell Engineering Division, cell bank (Tsukuba, Japan)^47^ and cultured following established protocols maintaining in a 1:1 mixture of EMEM (M2279, Merck) and Ham’s F-12 (N4888, Merck), supplemented with 5% fetal bovine serum (FBS; F7524, Merck), 5 µg/ml insulin, 10 ng/ml EGF and 1% penicillin-streptomycin. All cells were maintained at 37ºC in a humidified atmosphere with 5% CO_2_ in separate incubators. Upon reaching 80–90% confluence, accutase cell detachment solution (SCR005, Merck) or trypsin-EDTA 0.25% (Gibco™ 25200072) was used for the detachment of LSC and HCE-T cells during routine subcultures, respectively.

### Transfection

For experimental assays, cells were seeded in 6-well plates at a density of 2 × 10^5^ cells per well in growth medium. When the cells reached 60–70% confluence, transfection was conducted utilizing Lipofectamine™ RNAiMAX (13778150, Life Technologies, Thermo-Fisher), and following the manufacturer’s protocol. Transfection was done using either hsa-miR-204-5p miRCURY LNA miRNA Mimic (GeneGlobe ID - YM00471565-ADA Catalog No. − 339173, Qiagen) or the scrambled-sequence negative control (NC). Briefly, for each well, a transfection complex containing Lipofectamine and either miR-204-5p mimic or NC (30 pmol for all cell lines and 100 pmol for primary cells) in Opti-MeM^®^ medium were incubated for 5 minutes (20 min for primary cells) at room temperature and subsequently added dropwise onto the cells and allowed to incubate under standard culture conditions. At 24 h post-transfection (72 h for primary cells), total RNA was extracted and the expression level of target genes was detected using quantitative real‑time PCR (qRT-PCR). All transfection procedures were conducted in duplicate or triplicate to ensure consistency and reliability of results. For human primary LEC, we also investigated the effect of miR-204-5p overexpression (100 pmol) in an inflammatory environment using 2 µg/ml lipopolysaccharide (LPS) (Sigma-Aldrich, St. Louis, USA) added to the culture medium 48 h after transfection. Cells were then incubated overnight with LPS and then were collected for further analysis.

### miRNA toxicity assay

A fluorescent cell cytotoxicity assay was performed to determine the impact of miR-204-5p on cell viability. A live/dead staining assay was performed using 1mM Calcein-AM (Invitrogen, Thermo Fischer Scientific, Inc.) and Propidium Iodide (2 mg/ml) (PI: Sigma-Aldrich, Merck) to stain live and dead cells, respectively. Experiments were conducted in duplicate to assess the cell viability of T-LSC and mut-LSC. Briefly, cells were washed with Hanks Balance Salt Solution HBSS^+/+^ (ATCC, VA, USA) for 15 min at 37°C, and incubated with Calcein-AM green and PI red staining solution for 20 min at 37°C. Ultimately cells were washed with HBSS^+/+^ and imaged using EVOS^®^ FL Auto Imaging system (Life Technologies).

### Research animals and ethics

A total of 12 C57BL/6 male mice aged 6 weeks, as well as 6 129S1/SvImJ (WT-129S) and 12 129S1/SvImJ *Pax6*^*sey*/+^ mice (Het) were used in this study. The *Pax6*^*sey*/+^mice, developed by E. Simpson^28^ were obtained from the Mutant Mouse Resource Center at the University of Missouri. Both mouse strains were bred and housed in an air-conditioned environment at Linköping University animal facility, with constant temperature (21 ± 2°C) on a 12-hour light and dark cycle with free access to standard chow food and water. The animal experiments in this study were approved by the Linköping Regional Animal Ethics Committee (Approval no. 10940 − 2021) prior to the study and the procedures were performed in accordance with the Association for Research in Vision and Ophthalmology (ARVO) guidelines for the use of animals in Ophthalmic and Vision Research. The study is in accordance with ARRIVE guidelines. The animal euthanasia was carried out by cervical dislocation under deep anesthesia.

### *In vivo* corneal examinations

Using a Micron III rodent slit lamp camera (Phoenix Research Laboratories, USA), mouse corneas were initially visualized to examine the epithelial surface and the grade of transparency loss and corneal opacity^48^. Subsequently, corneas were imaged with a laser scanning in vivo confocal microscope (IVCM) (HRT3-RCM; Heidelberg Engineering, Heidelberg, Germany) to determine central epithelial phenotype, the status of inflammatory cells, and any possible invasion of blood vessels in the cornea.

### Murine LPS keratitis model

C57BL/6 mice were initially anesthetized by intraperitoneal injection of ketamine (50 mg/kg) (Orion Pharma AB, Sollentuna, Sweden) and xylazine (12 mg/kg) (Pfizer AB, Sollentuna, Sweden) with topical analgesia (Tetracaine) additionally given as eye drops. Using a 33-gauge needle (Hamilton Co., Reno, NV), 10 µg LPS^49^ and 60 pmol miR-204-5p or NC (with lipofectamine reagent) were injected subconjunctivally in the right eye while the contralateral eye was left untreated. After injection, both eyes were treated with Fucithalmic antibacterial ointment to minimize the possibility of secondary infection. miR-204-5p/NC (10 pmol) was thereafter topically administered 4 times daily for 2 days.

### *Pax6*^*Sey ****/***+^ small-eye mouse model of aniridia

WT-129S and Het mice on the 129S1/SvImJ background underwent anesthesia using a ketamine (50 mg/kg) / xylazine (12 mg/kg) mixture and to mitigate potential pain, Tetracaine was applied topically. To maintain precision and uniformity in treatment delivery, on the first day of treatment (day 0), both experimental and vehicle groups underwent a precise bilateral subconjunctival injection using lipofectamine reagent and miR-204-5p mimic and NC (120 pmol) respectively. Subsequently, from day 1 to day 6, four eye drops of miR-204-5p/NC with lipofectamine reagent were administered daily (20 pmol). On day 7 potential effects of miRNA delivery on the mouse corneas were evaluated using slit lamp microscopy. Mice were subsequently euthanized, and the corneal mRNA was extracted for quantitative verification of downstream gene targets.

### Total RNA (TRNA) extraction from cells and corneal tissues

Cells were harvested and TRNA was isolated 24- and 72-hours post-transfection for stem cells and LECs in the LPS model respectively using miRNeasy Tissue/Cells Advanced kit (from miR assays, 217604, Qiagen) or the GeneJET RNA Purification Kit (for transcriptomic assays, K0731, Life Technologies from Thermo-Fisher). For ex vivo experiments, after euthanizing the mice, eyes were promptly removed, washed in PBS, and then immersed in RNAlater solution (Qiagen, Hilden, Germany) for either immediate excision or temporarily stored at 4 ºC for subsequent cornea isolation. Each single harvested cornea per sample (non-pooled) was then lysed in lysis buffer (Qiagen, Hilden, Germany) and homogenized using a hand-held tissueRuptor (Qiagen, Hilden, Germany), on ice, and TRNA was extracted. TRNA was used for miRNA and mRNA complementary DNA (cDNA) and quantitative real‑time PCR. In the case of primary human LECs, RNA and protein were isolated using RNA/Protein isolation kit (DNA/RNA/Protein Purification Plus Micro Kit (Cat. Nr. 47700, Norgen Biotek CORP. Canada) following the manufacturer’s instructions.

### cDNA synthesis and Quantitative real‑time PCR (qRT-PCR) of miRNAs and mRNA from cells and corneal tissue samples

For cell lines and corneal tissues, the concentration of total RNA was detected using a Nanodrop spectrophotometer (Thermo Scientific, Inc.), and miRNA/mRNA cDNA was synthesized utilizing the TaqMan Advanced miRNA cDNA Synthesis Kit (A28007, Life Technologies from Thermo-Fisher) and SuperScript III Reverse Transcriptase (Invitrogen, Thermo Fisher Scientific, MA, USA) cDNA Synthesis Kit respectively according to manufacturer’s instructions. To evaluate the miR-204-5p expression level, we utilized TaqMan^®^ fast Advanced Master Mix and a TaqMan Advanced miRNA assay probe (A25576 Assay ID:478491_mir: hsa-miR-204-5p, Life Technologies from Thermo-Fisher). Reverse transcription for primary human LECs was performed using One Taq^®^ RT-PCR Kit (New England Biolabs INC, Frankfurt, Germany). For this purpose, 1 µg RNA was used as template and all the steps were performed as suggested by the manufacturer.

TaqMan qRT-PCR method was performed on a StepOnePlus Real-Time PCR System (Applied Biosystems, Thermo Fisher Scientific, Inc.) with U6 snRNA (4440887, Assay ID:001973: U6 snRNA, Life Technologies from Thermo-Fisher) as the endogenous control. For downstream gene expression analysis, qRT-PCR was conducted using PowerUp SYBR Green Master Mix (Applied Biosystems, Thermo Fisher Scientific, Inc.) using the protocol with appropriate gene-specific primers of 400 nM each with primer oligos obtained from Merck-Sigma and the following cycling parameters: 50°C for 2 min, 95°C for 2 min, and then 40 cycles of 95°C for 3 s and 60°C for 30 s. Both miRNA and mRNA qRT-PCR analyses were performed in triplicate to ensure reliability and consistency in the observed effects. Gene expression in primary LECs was evaluated using the SYBR Green kit (Qiagen N.V., Venlo, Netherlands) and primers were obtained from Qiagen (QuantiTect Primer Assay, Qiagen N.V., Venlo, Netherlands). Relative gene expression levels were determined using 2^− ΔΔCt ^method, normalized to an internal control gene (U6 or β-actin and TATA-binding protein (TBP) for primary cells) as the housekeeping reference gene. The primer sequences used are listed in Supplementary Table 1. The sequences for β-actin primers, which are commercially available by OriGene (human: HP204660, mouse: MP200232) and widely cited as housekeeping reference genes^50–52^, were further validated against the human genome using NCBI’s BLAST tool to be specific with no expected off-target products, and thus appropriate as reference housekeeping gene for the normalization of other targets. Pax6 primers were custom designed for both hPAX6 and mPax6, which we also validated via BLAST to be highly specific, with no expected off-target matches (amplicon size of 259 bp spanning exon 5 and 6 for human, amplicon size 106 bp spanning exon 4 and 5 for mouse) and thus optimal for qRT-PCR application. All other primers sequences are either commercially available products by OriGene or published in the literature for their specific targets, which we also validated via BLAST.

### Western Blot analysis from cells and tissues

To investigate changes in intracellular protein concentrations, we performed western blot immunostaining. First, scraped cells and the corneas were lysed on ice using RIPA lysis buffer supplemented with freshly added Halt™ Phosphatase/protease Inhibitor Cocktail with EDTA (78442, Thermo Scientific). Protein concentrations were then measured by Pierce™ BCA Protein Assay (A55864, Thermo Scientific) and 20 µg protein was loaded in 4–15% gradient Mini-PROTEAN_®_ TGX™ Precast Protein Gels (Bio-Rad #4561084). The proteins were transferred in a PVDF membrane and Tris-buffered saline (TTBS) (20 mM Tris–HCl [pH 7.4], 100 mM NaCl, and 0.1% Tween 20) including 5% non-fat dry milk for 1 h at room temperature and then incubated with primary antibodies (Supplementary Data. Table 2) at 4ºC overnight. The next day, the membranes were washed and then incubated for 2 h with the horseradish peroxidase (HRP)-labelled secondary antibodies (R&D system) (1:2000). Ultimately the bands were detected using enhanced chemiluminescence (ECL) western blotting substrate (Thermo Fisher #32106) and visualized using ImageQuant™ LAS 500 Chemiluminescent Imaging System (GE Healthcare, Waukesha, WI, US). ImageJ software was used to quantify the band intensities and results were normalized to β-actin expression.

### Statistical analysis

Statistical analysis was performed using GraphPad Prism software (Version 9.2.0, GraphPad Software, San Diego, CA, USA). Data are shown as mean ± SD and significances have been tested either by the independent t-test or one-way ANOVA followed by the Tukey test for multiple comparisons. Values have been normalized to the respective controls before comparison and p-values < 0.05 were considered statistically significant. Image analysis was performed using ImageJ software (ImageJ, NIH, USA)^53^ to assess and quantify cell viability where results were presented as a percentage of cell survival.

## Electronic supplementary material

Below is the link to the electronic supplementary material.

Supplementary Material 1

## Data Availability

The data supporting the findings of this study are available from the corresponding author upon request.
