# Supplementary material for: Effects of miR-204-5p modulation on PAX6 regulation and corneal inflammation
Source: Sci Rep. 2024 Nov 2;14:26436. doi: 10.1038/s41598-024-76654-w (PMC11531487; doi:10.1038/s41598-024-76654-w)
Supplement: Supplementary file 1 — Supplementary Material 1 [file 41598_2024_76654_MOESM1_ESM.docx]

**Supplementary Data.**

**Table 1. Primers used for qRT-PCR**

| Genes (Human/Mouse) | Primer | Sequence (5’-3’) |
| --- | --- | --- |
| *β-ACTIN (h)* | Forward  Reverse | CACCATTGGCAATGAGCGGTTC  AGGTCTTTGCGGATGTCCACGT |
| *β-actin (m)* | Forward  Reverse | CATTGCTGACAGGATGCAGAAGG  TGCTGGAAGGTGGACAGTGAGG |
| *ANGPT1 (h)* | Forward  Reverse | CAACAGTGTCCTTCAGAAGCAGC  CCAGCTTGATATACATCTGCACAG |
| *Angpt1 (m)* | Forward  Reverse | AACCGAGCCTACTCACAGTACG  GCATCCTTCGTGCTGAAATCGG |
| *VEGFA (h)* | Forward  Reverse | CACTAACAGCACATCTGGAGACC  TGAGCACAAGGAGCAGCGTAGA |
| *Vegfa (m)* | Forward  Reverse | CTGCTGTAACGATGAAGCCCTG  GCTGTAGGAAGCTCATCTCTCC |
| *IL-1beta (m)* | Forward  Reverse | TTCAGGCAGGCAGTATCACTC  GAAGGTCCACGGGAAAGACAC |
| *TNF-alpha (m)* | Forward  Reverse | CCCTCACACTCAGATCATCTTCT  GCTACGACGTGGGCTACAG |
| *PAX6 (h)* | Forward  Reverse | TGAATCAGCTCGGTGGTGTC  CACTCCCGCTTATACTGGGC |
| *Pax6 (m)* | Forward  Reverse | ACTTCAGTACCAGGGCAAC  GAGCTTCATCCGAGTCTTCTTC |

**Table 2. Primary antibodies used for Western Blot**

| **Antibody** | **Dilution** | **Cat. No.** | **Manufacturer** |
| --- | --- | --- | --- |
| VEGFA Rabbit PolyAb | 1:1000 | 19003-1-AP | Proteintech, Martinsried, Germany |
| Angpt1 Rabbit PolyAb | 1:1000 | 27093-1-AP | Proteintech, Martinsried, Germany |
| PAX6 mouse PolyAb | 1:1000 | AB2237 | Millipore, Watford, UK |
| β-actin Rabbit PolyAb | 1:10000 | Ab8227 | Abcam, Cambridge, UK |
| pERK1/2 Rabbit PolyAb | 1:1000 | 36-8800 | Invitrogen, M.A, USA |

**
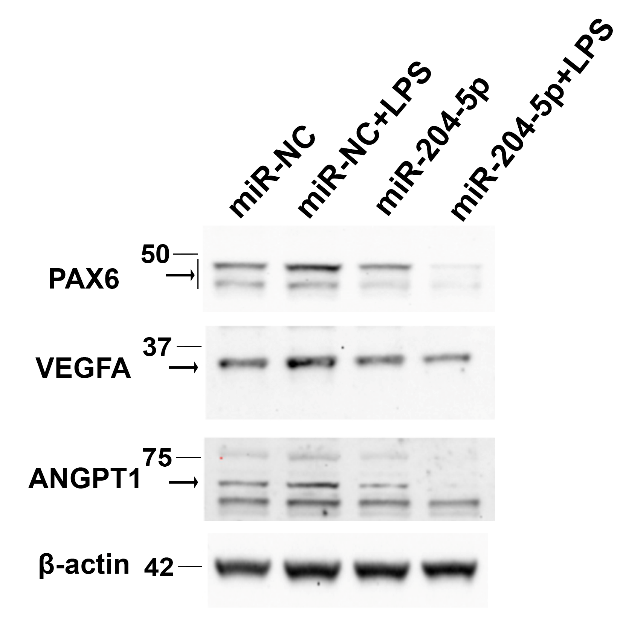
**

**Supplementary Figure 1.** Representative western blot images (cropped from the original blot indicated in supplementary figure 3. A-C) of one of five donors. No significant changes in PAX6 protein level were observed after LPS stimulation of LEC cells that were treated with miR-204-5p. VEGFA was not significantly suppressed, while the reduction in ANGPT1 level was significant with miR-20r-5p treatment both with and without LPS stimulation.

**
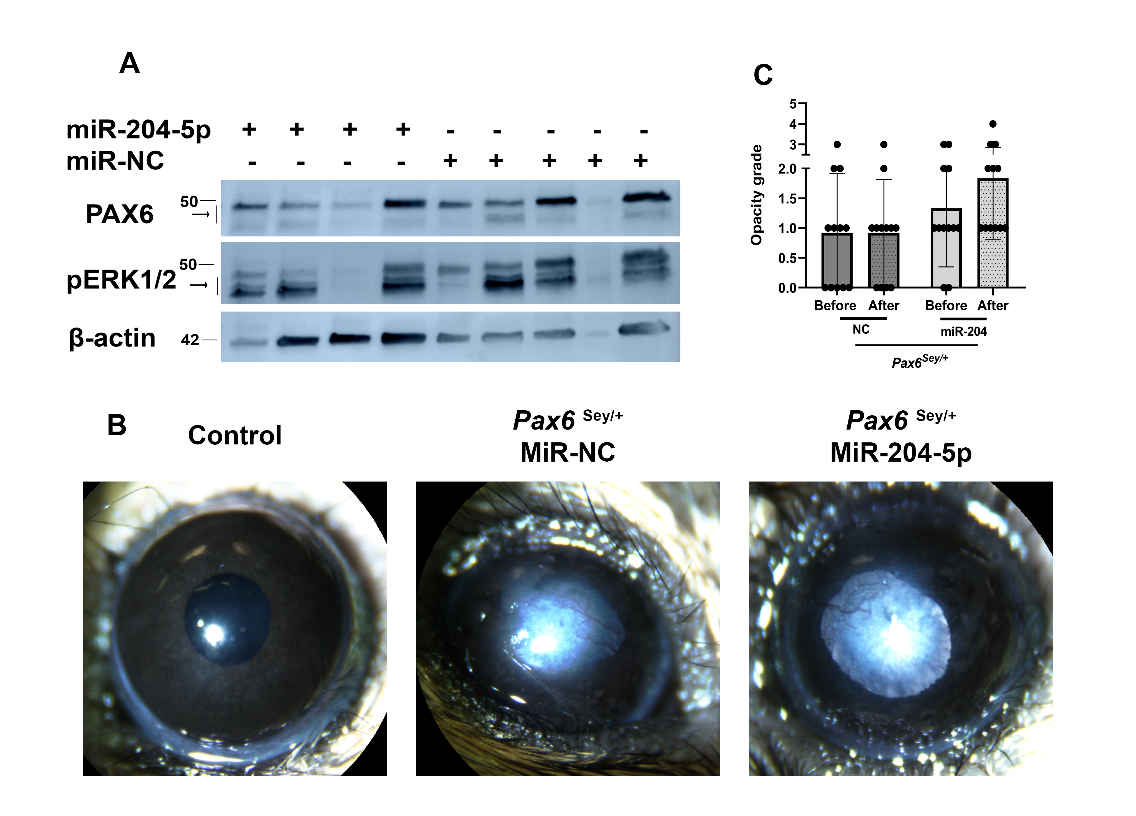
**

**Supplementary Figure 2. Western blot and Slit lamp analysis indicated no alterations between the two groups of aniridia mice being treated with either miR-204-5p or NC.** (A) Western blot images indicating PAX6 and ERK1/2 expression after the corneas were administered with either miR-204-5p or miR-NC. (Images are cropped from the original western blot images indicated in supplementary Figure 3.D-F). Following miRNA treatment, phosphorylated ERK 1/2 did not significantly change (p = 0.4), and no alteration in PAX6 level was observed in miR-204-5p or NC-treated groups. (B) There was no notable difference in the opacity and vascularization status of *Pax6*^Sey/+^ corneas following 7 days of miR-204-5p topical administration. (C) Statistical analysis did not reveal any noticeable alteration in corneal grade opacity in either group.


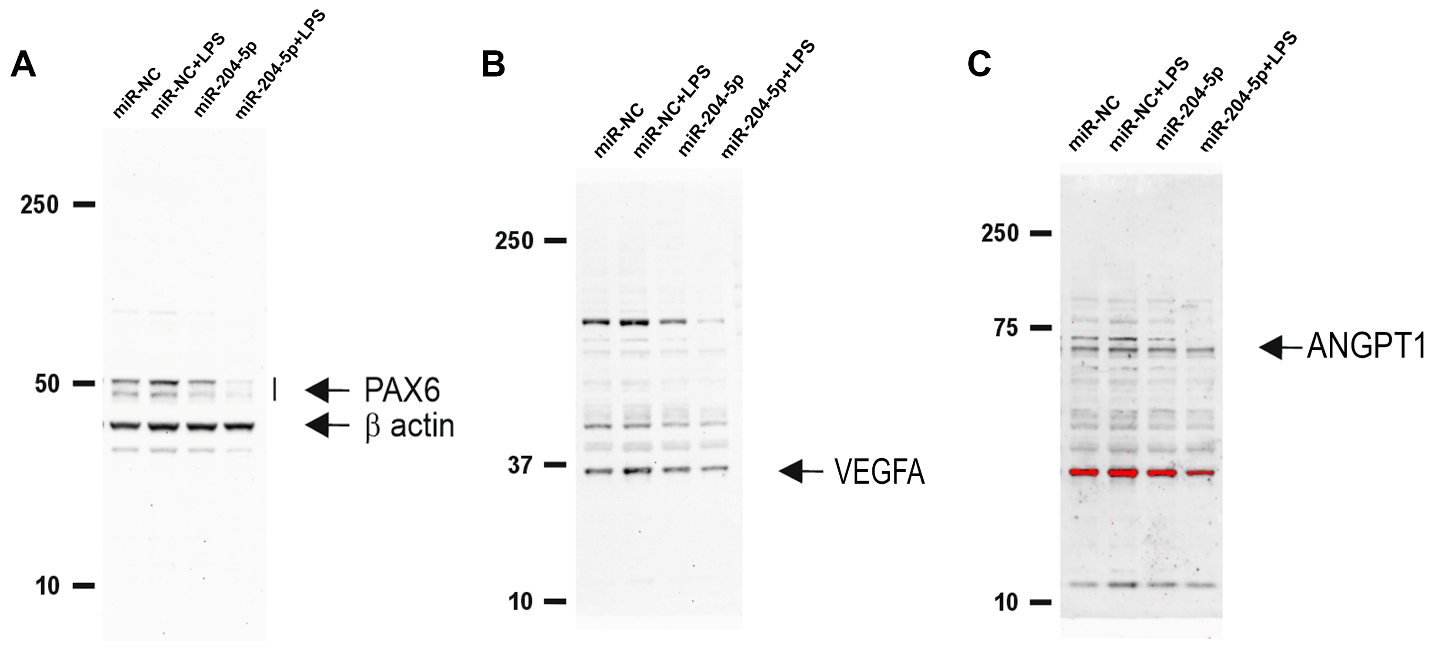


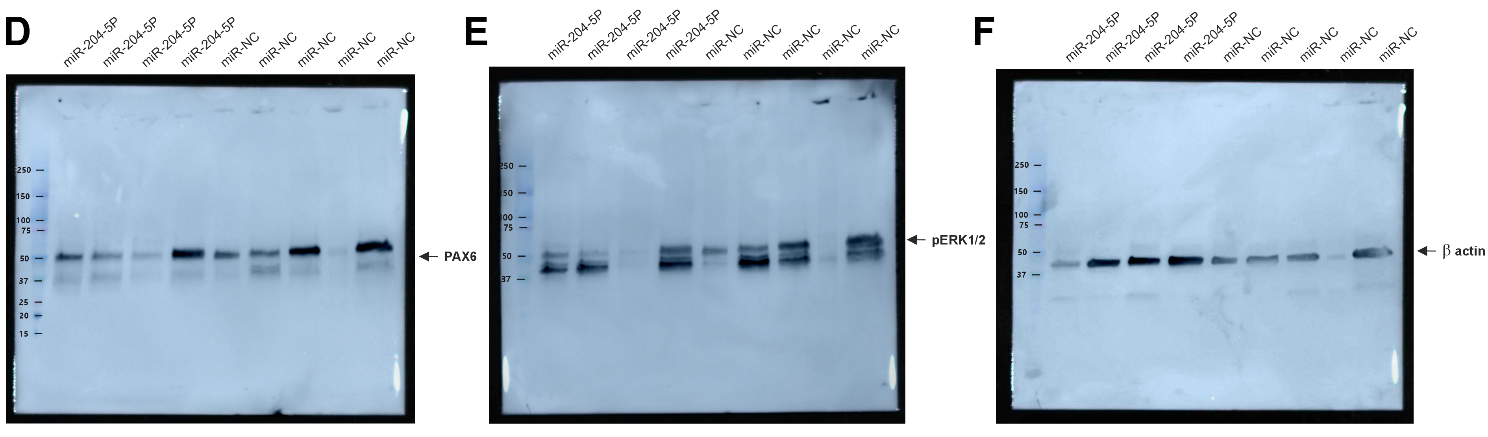


**Supplementary Figure 3.** Original, uncropped Western blot images corresponding to Supplementary Figure 1 (A-C) and Supplementary Figure 2 (D-F).
